# Fermented Porcine Placenta and Its Dipeptides Modulate Cellular Senescence in Human Keratinocytes

**DOI:** 10.3390/cimb47110941

**Published:** 2025-11-12

**Authors:** Yea Jung Choi, Minseo Kang, Mu Hyun Jin, Jongbae Kim, Won Kyung Lee, Seok-Seon Roh, Ki Sung Kang, Gwi Seo Hwang, Sangki Park, Sullim Lee

**Affiliations:** 1 College of Korean Medicine, Gachon University, Seongnam 13120, Republic of Korea; domdada22@gachon.ac.kr (Y.J.C.); kkang@gachon.ac.kr (K.S.K.);; 2 Department of Life Science, College of Bio-Nano Technology, Gachon University, Seongnam 13120, Republic of Korea; bana825@gachon.ac.kr; 3 Science Research Park, LG Household and Healthcare Ltd., Seoul 07795, Republic of Korea; mhjin@lghnh.com (M.H.J.); jbkim@lghnh.com (J.K.); wksophie@lghnh.com (W.K.L.); rssdr@hanmail.net (S.-S.R.)

**Keywords:** fermented porcine placenta, Leu–Gly, Pro–Hyp, keratinocyte senescence, oxidative stress, NAD^+^ metabolism, mitochondrial regulation

## Abstract

Skin aging is primarily driven by oxidative stress, mitochondrial dysfunction, and cell cycle dysregulation. This study investigated the anti-senescence effects of fermented porcine placenta (FPP) and its dipeptides, leucine–glycine (LG) and proline–hydroxyproline (PH), in human epidermal keratinocytes (HEKs), using nicotinamide mononucleotide (NMN) as a reference for nicotinamide adenine dinucleotide (NAD^+^)-related pathways. FPP suppressed senescence-associated β-galactosidase (SA-β-gal) activity and Cyclin-dependent kinase inhibitor 2A (p16) expression while enhancing adenosine triphosphate (ATP) production and sirtuin 1 (SIRT1)–peroxisome proliferator-activated receptor-gamma coactivator 1α (PGC-1α) signaling. LG and PH exhibited distinct actions: LG improved redox balance by increasing the NAD^+^/NADH ratio and NAD(P)H quinone oxidoreductase 1 (NQO1) activity, whereas PH modulated cell cycle regulators and upregulated sirtuin 3 (SIRT3) expression. Although both peptides contributed to FPP’s effects, their combination did not fully replicate its overall activity, suggesting synergistic roles of multiple bioactive constituents. These findings highlight FPP as a multifactorial modulator of keratinocyte senescence, acting via mitochondrial and redox-related mechanisms.

## 1. Introduction

The skin, the body’s largest organ, functions as the primary barrier against environmental stressors such as ultraviolet (UV) radiation, air pollutants, and oxidative agents, rendering it highly susceptible to premature aging [1,2]. The epidermis, composed mainly of keratinocytes, maintains structural integrity and immune defense by producing barrier proteins, including keratins, involucrin, and loricrin, and by secreting cytokines, such as interleukin (IL)-6, and interleukin (IL)-1β to regulate inflammatory and homeostatic responses [3,4,5]. Chronic exposure to environmental insults accelerates oxidative damage and mitochondrial dysfunction, leading to cellular senescence through impaired adenosine triphosphate (ATP) production and activation of senescence-associated genes, such as Cyclin-dependent kinase inhibitor 2A (p16) and Cyclin-Dependent Kinase Inhibitor 1A (p21) [6,7,8,9,10,11]. These processes are mechanistically linked to the dysregulation of nicotinamide adenine dinucleotide (NAD^+^) metabolism and sirtuin signaling, both of which are essential for maintaining mitochondrial homeostasis and redox balance.

Placental extracts have long been investigated for their regenerative and protective roles in dermatology. Studies have shown that porcine placental extracts and exosomes derived from placental extract–treated mesenchymal stem cells protect dermal fibroblasts from ultraviolet (UV)- or glycation-induced injury by enhancing proliferation and reducing reactive oxygen species (ROS) accumulation [12]. Moreover, fermentation of porcine placenta with probiotic strains such as *Enterococcus faecalis* PR31 has been reported to enhance its biological activity, improve skin hydration, and produce metabolites, including lactic acid, pyruvic acid, ornithine, and tyramine, which contribute to antioxidant and moisturizing effects [13]. These observations suggest that fermentation enhances the bioactivity of placental preparations. However, the molecular mechanisms by which fermented placenta exerts protective effects on keratinocytes remain largely unexplored.

A decline in NAD^+^ levels is a hallmark of aging across multiple tissues, and continuous UV exposure further accelerates NAD^+^ depletion via Deoxyribonucleic acid (DNA) repair processes [14,15,16,17]. Restoration of NAD^+^ via the nicotinamide phosphoribosyltransferase (NAMPT) salvage pathway or supplementation with NAD^+^ precursors, such as nicotinamide mononucleotide (NMN) and nicotinamide riboside (NR), has been shown to protect keratinocytes by activating the Sirtuin 1 (SIRT1)–Tumor Protein P53 (p53) axis and improving mitochondrial performance [18,19]. In addition, antioxidant enzymes, such as NAD(P)H quinone oxidoreductase 1 (NQO1), maintain redox balance by oxidizing Reduced Nicotinamide Adenine Dinucleotide (NADH) to NAD^+^, thereby limiting oxidative damage and sustaining cellular metabolism [20,21]. Given that SIRT1 and Sirtuin 3 (SIRT3) regulate mitochondrial biogenesis and energy homeostasis through Peroxisome Proliferator-Activated Receptor Gamma Coactivator 1-alpha (PGC-1α), and transcription factor A(TFAM) [22,23,24,25], compounds capable of modulating these targets may provide multifaceted protection against skin aging.

FPP mainly consists of lysine, leucine, arginine, alanine, proline, and other amino acids, among which leucine, lysine, and proline account for 3.02%, 2.12%, and 0.7%, respectively [26]. Although the quantitative proportion of dipeptides was not determined in this analysis, previous studies have identified leucine–glycine (LG) and proline–hydroxyproline (PH) as bioactive peptides produced during the fermentation of porcine placenta, which may contribute to its biological effects. Recent reports further suggest that these small peptides derived from FPP, including LG and PH, exert distinct biological functions. Leucine activates the AMP-activated protein kinase (AMPK)–SIRT1–PGC-1α signaling axis, thereby enhancing mitochondrial activity and maintaining redox homeostasis [27,28]. Hydroxyproline-containing dipeptides, such as PH, which are collagen-derived metabolites, have been implicated in mitochondrial regulation via SIRT3-mediated antioxidant defense [29]. Collectively, these findings indicate that LG may support NAD^+^ metabolism and redox stability, whereas PH may modulate the mitochondrial pathways associated with cellular senescence. However, direct mechanistic comparisons between FPP and its peptide derivatives in epidermal keratinocytes remain to be explored.

This study highlights the novelty of directly comparing the mechanisms of action of FPP and its representative dipeptides, LG and PH, in human keratinocytes, providing new insights into the modulation of cellular senescence by FPP-derived peptide components. Therefore, this study aimed to elucidate the cellular mechanisms underlying the anti-senescence effects of FPP and its major dipeptide components, LG and PH, in human epidermal keratinocytes (HEKs). Multiple parameters, including senescence-associated β-galactosidase (SA-β-gal) activity, p16 and p21 expression, intracellular ROS levels, NAD^+^/NADH ratio, ATP production, NQO1 activity, and mitochondrial regulators (SIRT1, SIRT3, and PGC-1α), were analyzed to characterize the distinct and overlapping actions of FPP and its metabolites. These findings provide mechanistic insight into how FPP and its dipeptides modulate oxidative stress and mitochondrial homeostasis, contributing to cellular protection against skin aging.

## 2. Materials and Methods

### 2.1. Sample Preparation

Leucine–Glycine (LG; Cat# G-2495) and Proline–Hydroxyproline (PH; Cat# G-3025) were purchased from Bachem AG (Bubendorf, Switzerland). Fermented placenta extract (FPP) was provided by HORUS Co., Ltd. (Tokyo, Japan). All samples were dissolved in phosphate–buffered saline (PBS; Takara, Kusatsu, Japan) prior to treatment in the cell experiments.

### 2.2. Cell Culture

Primary HEKs (PromoCell GmbH, Cat# C-12003, Heidelberg, Germany) were cultured in Dulbecco’s Modified Eagle Medium (DMEM; Corning, Cat# 10-013-CV, Manassas, VA, USA) supplemented with 10% fetal bovine serum (FBS; Atlas, Cat# F-0500-A, Fort Collins, CO, USA) and 1% penicillin–streptomycin (Gibco, Cat# 15140122, Grand Island, NY, USA). Cells were maintained at 37 °C in a humidified incubator with 5% CO_2_ to support optimal growth and physiological conditions of the cells. All assays were performed in triplicate as technical replicates and were independently repeated using separate cell cultures as biological replicates.

### 2.3. SA-β-Galactosidase Staining Assay

Cellular senescence was evaluated using a Senescence β-Galactosidase Staining Kit (ab65351; Abcam, Cambridge, UK) according to the manufacturer’s protocol. HEKs were cultured in 6-well plates and treated with the indicated samples. After treatment, the cells were rinsed with PBS and fixed with 0.5 mL of Fixative Solution III for 10–15 min at room temperature. A fresh staining solution was prepared by mixing Staining Solution II, 100× Staining Supplement, and X-gal solution (20 mg/mL in DMSO; Sigma-Aldrich, Burlington, Massachusetts, USA) at the recommended ratios, and 0.5 mL of this solution was added to each well. The plates were incubated overnight at 37 °C without CO_2_, and the formation of blue precipitates, indicating SA-β-gal activity, was observed under a light microscope at 200× magnification.

### 2.4. Western Blotting

HEKs were seeded in flat-bottom 6-well plates at a density of 3 × 10^5^ cells/well and incubated for 24 h. To induce starvation, the cells were cultured in serum-free medium for an additional 24 h and then treated with FPP, LG, PH, or NMN for 24 h. After treatment, the cells were harvested to evaluate the expression of sirtuin 1 (SIRT1), sirtuin 3 (SIRT3), peroxisome proliferator-activated receptor gamma coactivator 1-alpha (PGC-1α), p16, p21, and GAPDH.

Cells were lysed in 1× radioimmunoprecipitation assay (RIPA) buffer (Tech & Innovation, Chuncheon, Republic of Korea), and the soluble fractions were collected as the protein samples. Protein concentrations were quantified using a bicinchoninic acid (BCA) assay kit (Merck, Rahway, NJ, USA). Equal amounts of protein were separated by SDS-PAGE and transferred onto membranes, which were incubated with primary antibodies against SIRT1, SIRT3, PGC-1α, p16, p21, and GAPDH (Cell Signaling Technology, Danvers, MA, USA) for 4 h at room temperature. After washing, the membranes were incubated with horseradish peroxidase-conjugated goat anti-rabbit IgG secondary antibodies (Cell Signaling Technology, Danvers, MA, USA) for 1 h at room temperature. Protein bands were visualized using SuperSignal West Femto Maximum Sensitivity Chemiluminescent Substrate (Thermo Fisher Scientific, Waltham, MA, USA) and detected using the Fusion Solo Chemiluminescent System (PEQLAB Biotechnologie GmbH, Erlangen, Germany).

### 2.5. ROS Assay

HEKs were seeded in black, clear-bottom 96-well plates (1.0 × 10^4^ cells/well) and incubated overnight. Cells were loaded with 10 μM DCF-DA for 30 min at 37 °C in the dark, washed with PBS (Takara, Kusatsu, Japan), and treated with FPP (25, 50, 100 μg/mL), LG (1, 5, 25 μg/mL), PH (1, 5, 25 μg/mL), or NMN (100 μM) for 30 min. Untreated cells were used as controls, and tBHP (Sigma-Aldrich, Burlington, MA, USA); 100 μM, 15 min) was used as the positive control. Intracellular ROS levels were measured using a DCF-DA (Sigma-Aldrich, Burlington, MA, USA) fluorescence assay (Abcam, Cambridge, UK; ab113851). Fluorescence (Ex/Em = 485/535 nm) was measured using a microplate reader, and the values were expressed relative to those of untreated controls.

### 2.6. NAD^+^/NADH Assay

HEKs were seeded at a density of 3 × 10^5^ cells per well in 6-well plates and cultured for 24 h. To induce starvation, the culture medium was replaced with serum-free medium and incubated for an additional 24 h. Cells were then treated with the indicated concentrations of samples for 24 h, after which the cell extracts were harvested. Intracellular NAD^+^ and NADH levels were determined using an NAD/NADH Assay Kit (Colorimetric, Abcam, Cambridge, UK, Cat# ab65348) according to the manufacturer’s instructions. Absorbance was measured at 450 nm using a microplate reader at 30 min intervals for 1–4 h.

### 2.7. Measurement of NQO1 Activity

HEKs were seeded at a density of 3 × 10^5^ cells per well in 6-well plates and incubated for 24 h. The medium was then replaced with serum-free medium to induce starvation for an additional 24 h, followed by treatment with the indicated samples for 24 h. After treatment, the cells were washed twice with PBS, harvested, and pelleted by centrifugation (500× *g* for 5 min at 4 °C). NQO1 activity was measured using an NQO1 Activity Assay Kit (Abcam, Cambridge, UK, Cat# ab184867) according to the manufacturer’s protocol. Absorbance was recorded at 440 nm using a microplate reader at 20 s intervals for 5 min.

### 2.8. Statistical Analyses

Statistical evaluations were conducted using GraphPad Prism (v. 8.0.1; GraphPad Software Inc., La Jolla, CA, USA). Differences among experimental groups were analyzed using one-way ANOVA, and Tukey’s multiple comparison test was used for post hoc analysis. Statistical significance was set at *p* < 0.05.

## 3. Results

### 3.1. β-Galactosidase Activity as a Marker of Cellular Senescence

**Figure 1:**
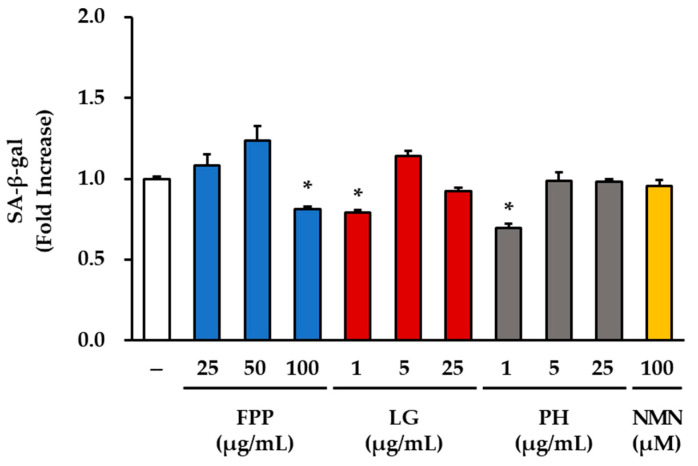
Effects of FPP, LG, PH, and NMN on β-galactosidase activity in HEKs. HEKs were seeded at a density of 3 × 10^5^ cells per well in 6-well plates and treated with FPP (25, 50, and 100 μg/mL), LG (1, 5, and 25 μg/mL), PH (1, 5, and 25 μg/mL), or NMN (100 μM) for 24 h. Senescence-associated β-galactosidase activity was measured using a commercial staining kit and quantified by measuring the cellular staining intensity. Data are expressed as mean ± SEM (*n* = 3). Statistical significance was determined using one-way ANOVA followed by Tukey’s post hoc test. * *p* < 0.05 vs. untreated control.

A β-galactosidase activity assay was performed to evaluate the effects of FPP, LG, PH, and NMN on cellular senescence (Figure 1). Treatment with FPP at the highest concentration (100 μg/mL) led to a statistically significant reduction in β-galactosidase activity (0.78 ± 0.02-fold relative to the control) compared to the untreated group (*p* < 0.05). In contrast, treatment with FPP at 25 and 50 μg/mL did not significantly change the levels of β-galactosidase activity, which were similar to or slightly higher than those of the untreated control.

A significant decrease in β-galactosidase activity was observed only at the lowest concentration of LG (1 μg/mL, *p* < 0.05), whereas no significant differences were observed at higher concentrations (5 and 25 μg/mL). Similarly, PH treatment significantly reduced β-galactosidase activity at 1 μg/mL (*p* < 0.05); however, this effect was not evident at 5 or 25 μg/mL, where the activity remained comparable to that of the untreated control group. These results indicate that both LG and PH exert inhibitory effects on β-galactosidase activity only at low concentrations, with no significant effect at higher concentrations. In the NMN-treated group (100 μM), β-galactosidase activity was not significantly different from that of the control group.

Collectively, these results demonstrate that FPP exerts a measurable reduction in β-galactosidase activity only at high concentrations, whereas LG and PH are effective at lowering β-galactosidase activity at the lowest concentration tested but not at higher concentrations. NMN treatment did not affect β-galactosidase activity in the present study.

### 3.2. p16 and p21 Expression as Indicators of Cellular Senescence

**Figure 2:**
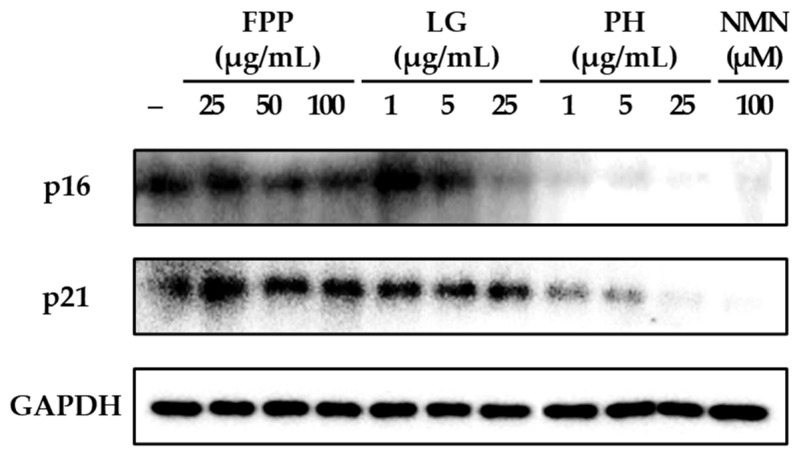

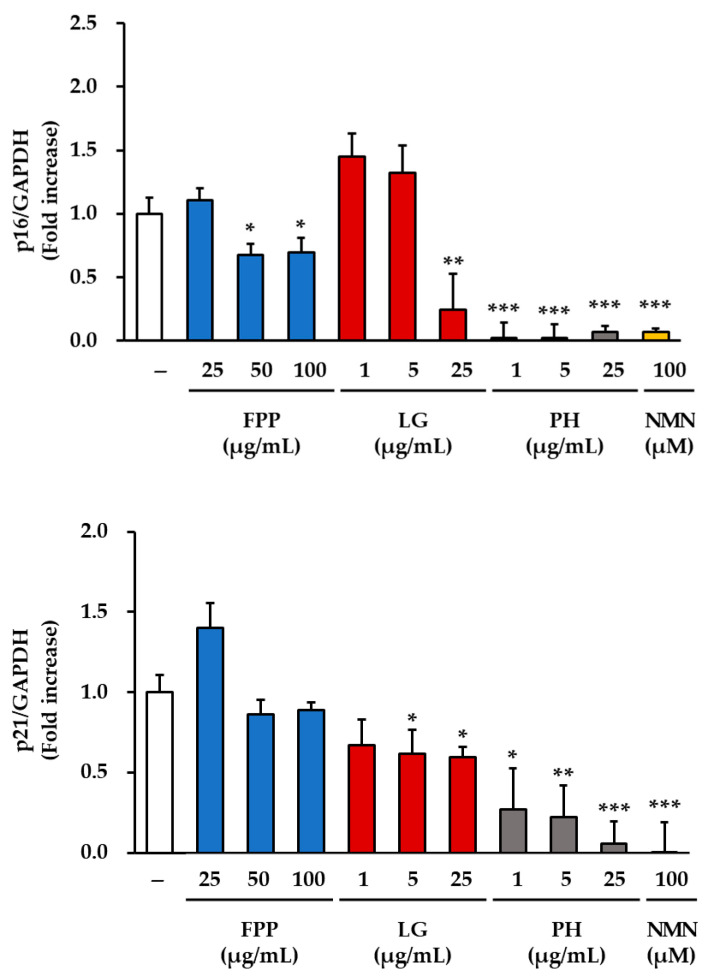
Effects of FPP, LG, PH, and NMN on p16 and p21 protein expression in HEKs. HEKs were treated with FPP (25, 50, and 100 μg/mL), LG (1, 5, and 25 μg/mL), PH (1, 5, and 25 μg/mL), or NMN (100 μM) for 24 h. The protein expression levels of p16 and p21 were determined by Western blot analysis and normalized to GAPDH. Quantitative data are presented as mean ± SEM (*n* = 3). Statistical significance was evaluated using one-way analysis of variance (ANOVA), followed by Tukey’s post hoc test. * *p* < 0.05, ** *p* < 0.01, and *** *p* < 0.001 vs. untreated control.

The expression levels of the cell cycle regulatory protein p16 were markedly altered following treatment with FPP, LG, PH, and NMN (Figure 2). In the FPP-treated group, p16 protein expression was reduced in a concentration-dependent manner. Although a decrease was observed at all tested concentrations (25, 50, and 100 μg/mL), statistically significant inhibitory effects were detected specifically at 50 and 100 μg/mL (*p* < 0.05), indicating that higher doses of FPP are required to achieve consistent suppression of p16 expression compared with the untreated control group.

LG treatment exhibited a biphasic pattern. At lower concentrations (1 and 5 μg/mL), p16 expression showed a slight increasing trend relative to that in the control, suggesting a potential stimulatory response at low doses. However, at the highest concentration tested (25 μg/mL), LG resulted in a significant reduction in p16 expression (*p* < 0.01). This indicates that LG exerts an inhibitory effect on p16 expression only at higher concentrations, whereas the effect at lower concentrations remains negligible or slightly opposite.

In contrast, PH exhibited a clear and consistent inhibitory effect at all tested concentrations. Significant suppression of p16 expression was observed at 1, 5, and 25 μg/mL, with strong statistical significance at all concentrations (*p* < 0.001), indicating that PH exerts a potent and dose-independent inhibitory effect on this senescence-associated protein. Similarly, NMN (100 μM) treatment exhibited the most pronounced reduction in p16 expression among all groups, with a highly significant inhibition (*p* < 0.001).

Analysis of p21 protein expression revealed distinct patterns compared with those of p16. In the FPP-treated group, p21 expression remained comparable to that of the untreated control, with no statistically significant differences observed at any of the tested concentrations. In contrast, LG gradually inhibited p21 expression in a dose-dependent manner, with significant reductions observed at 5 and 25 μg/mL (*p* < 0.05). PH demonstrated strong inhibitory activity, significantly reducing p21 expression at all tested concentrations, with a clear concentration-dependent trend (*p* < 0.05, *p* < 0.01, *p* < 0.001). NMN treatment also resulted in a robust reduction in p21 expression, with a highly significant inhibition (*p* < 0.001).

Collectively, these findings indicate that FPP selectively suppresses p16 expression only at higher concentrations and has little effect on p21. In contrast, LG displayed differential effects, increasing p16 expression at low concentrations but suppressing both p16 and p21 expressions at higher concentrations. PH consistently and strongly suppressed both p16 and p21 expression, regardless of concentration, and NMN exerted the most potent inhibitory effects on both proteins under the experimental conditions used.

### 3.3. ROS Levels as an Indicator of Oxidative Stress

**Figure 3:**
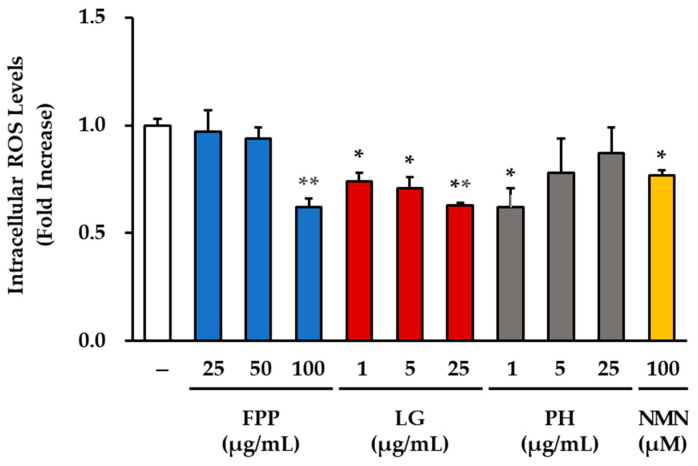
Effects of FPP, LG, PH, and NMN on intracellular ROS levels in HEKs. HEKs were treated with FPP (25, 50, and 100 μg/mL), LG (1, 5, and 25 μg/mL), PH (1, 5, and 25 μg/mL), or NMN (100 μM) for 30 min. Intracellular ROS levels were measured using the DCF-DA assay and quantified by fluorescence intensity. Data are expressed as mean ± SEM (*n* = 3). Statistical significance was evaluated using one-way ANOVA followed by Tukey’s post hoc test. * *p* < 0.05, ** *p* < 0.01 vs. untreated control.

Intracellular ROS levels were measured to evaluate the antioxidant effects of FPP, LG, PH, and NMN in HEKs (Figure 3). In the FPP-treated groups, a significant reduction in ROS generation was observed only at the highest concentration of 100 μg/mL (*p* < 0.01), whereas 25 and 50 μg/mL FPP treatments showed no statistically significant changes compared with the untreated control.

In the LG-treated groups, the ROS levels were markedly reduced in a concentration-dependent manner. At 1 and 5 μg/mL, ROS generation decreased significantly (*p* < 0.05), and further reductions were observed at 25 μg/mL (*p* < 0.01), indicating a strong and consistent antioxidant effect of LG at all concentrations.

PH treatment resulted in a selective effect, with significant suppression of ROS levels detected only at 1 μg/mL (*p* < 0.05), whereas no differences were observed at 5 and 25 μg/mL compared to the untreated group.

NMN treatment (100 μM) also significantly reduced ROS levels (*p* < 0.01), although the magnitude of suppression was less pronounced than that observed in the FPP, LG, and PH-treated groups.

Collectively, these results demonstrate that FPP exerts ROS-reducing activity only at high concentrations, whereas LG consistently and robustly decreases ROS generation at all tested concentrations. PH exhibited antioxidant effects only at low concentrations, and NMN treatment moderately reduced ROS levels, supporting its role as an indirect modulator of oxidative stress.

### 3.4. NAD^+^/NADH Ratio as an Indicator of Cellular Redox Balance

**Figure 4:**
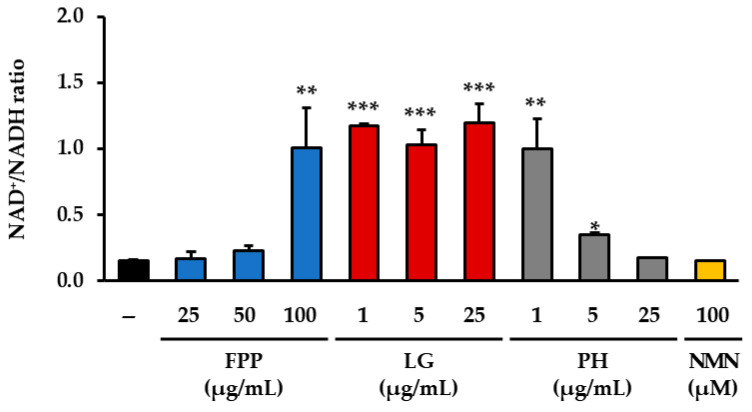
Effects of FPP, LG, PH, and NMN on NAD^+^/NADH ratio. The NAD^+^/NADH ratio was determined using a commercial NAD^+^/NADH Assay Kit following the manufacturer’s protocol, and absorbance was measured at 450 nm using a microplate reader. Data are expressed as the mean ± SEM (*n* = 3). Statistical significance was assessed using one-way ANOVA with Tukey’s post hoc test. * *p* < 0.05, ** *p* < 0.01, *** *p* < 0.001 vs. untreated control.

The measurement of the intracellular NAD^+^/NADH ratio revealed distinct changes depending on the treatment conditions (Figure 4). In the FPP-treated groups, a significant increase in the NAD^+^/NADH ratio was observed only at the highest concentration tested (100 μg/mL), whereas no significant alterations were detected at 25 or 50 μg/mL when compared with the untreated control group. In the LG–treated groups, a clear elevation in the NAD^+^/NADH ratio was consistently observed across all tested concentrations, with statistical significance at each level, indicating a reproducible effect of this compound on the cellular redox balance. PH treatment showed a more restricted pattern of activity, with significant increases in the NAD^+^/NADH ratio detected only at the lower concentrations of 1 and 5 μg/mL, whereas no significant changes were evident at 25 μg/mL.

In contrast, NMN treatment (100 μM), despite being a known positive control for NAD^+^ metabolism, did not lead to a statistically significant change in the NAD^+^/NADH ratio relative to the untreated control group under the present experimental conditions. These results collectively demonstrate that the modulation of the NAD^+^/NADH ratio by FPP and PH occurs only within specific concentration ranges, whereas LG exerts a more consistent and robust effect across a wider range of concentrations.

### 3.5. ATP Production as an Indicator of Cellular Energy Metabolism

**Figure 5:**
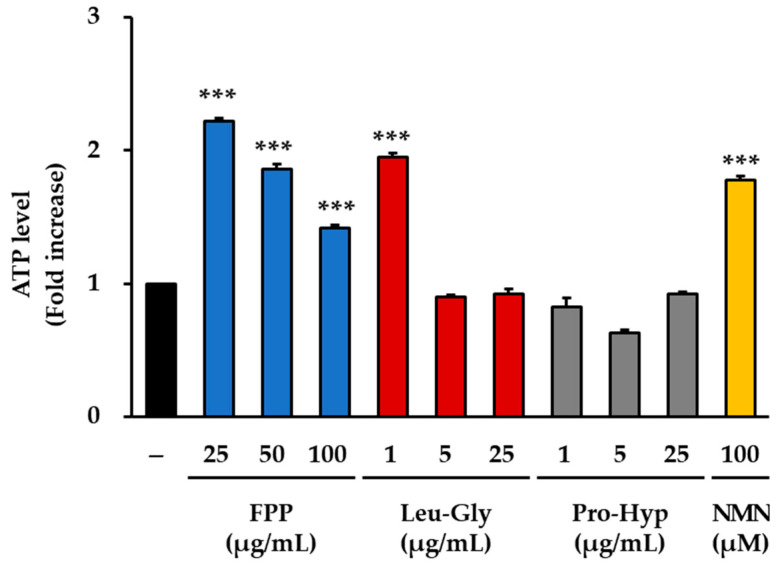
Effects of FPP, Leu-Gly, Pro-Hyp, and NMN on intracellular ATP levels in HEKs. Intracellular ATP levels were determined using a fluorometric ATP Assay Kit. HEKs were treated with FPP (25, 50, and 100 μg/mL), Leu-Gly (1, 5, and 25 μg/mL), Pro-Hyp (1, 5, and 25 μg/mL), or NMN (100 µM) for 24 h. Cell lysates were prepared and deproteinized to remove interfering enzymes before analysis. Data are expressed as the mean ± SEM (*n* = 2). Statistical significance was assessed using one-way ANOVA followed by Tukey’s post hoc test. *** *p* < 0.001 vs. untreated control.

Measurement of intracellular ATP production revealed clear differences among the treatment groups (Figure 5). In the FPP-treated cells, ATP concentrations were significantly elevated at all tested concentrations (25, 50, and 100 μg/mL) compared to those in the untreated control group (*p* < 0.001). The most pronounced increase was observed at 25 μg/mL, which represented the peak effect in this treatment group. Although the magnitude of the increase was slightly lower at 50 and 100 μg/mL than at 25 μg/mL, ATP concentrations at both doses remained significantly higher than those in the control group, indicating a consistent effect of FPP across all tested concentrations.

LG treatment resulted in different response patterns. A statistically significant increase in ATP concentration was observed only at the lowest concentration tested (1 μg/mL, *p* < 0.001). At higher concentrations (5 and 25 μg/mL), ATP levels did not differ significantly from those of the untreated control, and the overall effect was minimal. This concentration-dependent response suggests that LG exerts measurable effects on cellular ATP production only at low doses, whereas higher doses do not induce additional changes.

In the PH–treated groups (1, 5, and 25 μg/mL), no statistically significant increase in ATP concentration was detected. Across all concentrations tested, ATP levels remained comparable to or, in some cases, slightly lower than those of the untreated control group. These results indicate that PH did not contribute to the enhancement of ATP production under the conditions used in this study.

In contrast, 100 μM NMN treatment significantly increased intracellular ATP concentration compared to that in the control group (*p* < 0.001). The effect observed in the NMN-treated group was consistent with its role as a precursor of NAD^+^, which is closely linked to cellular energy metabolism.

Collectively, these findings demonstrate that FPP can increase ATP production at all tested concentrations, with the strongest effect observed at 25 μg/mL, whereas LG is effective only at low concentrations. PH did not exert significant effects on ATP production, whereas NMN treatment resulted in a robust increase, confirming its expected role in promoting energy metabolism.

### 3.6. NQO1 Activity as an Indicator of Antioxidant Response

**Figure 6:**
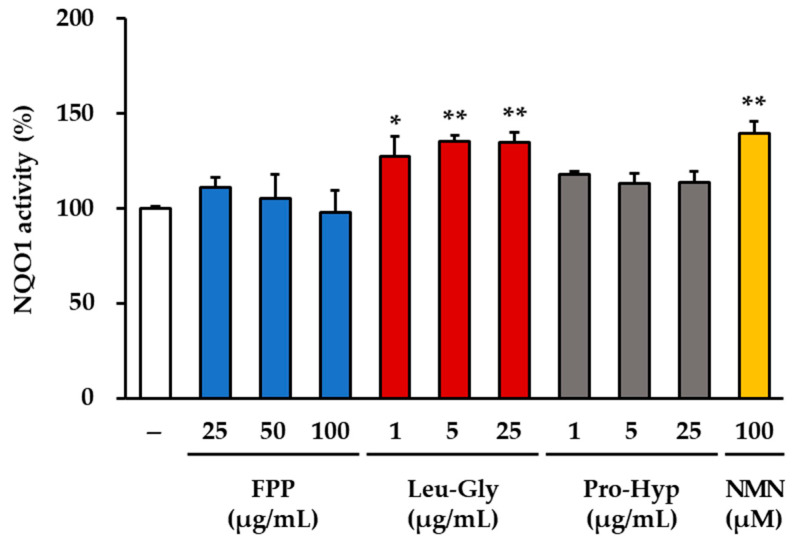
Effects of FPP, LG, PH, and NMN on NQO1 activity in HEKs. HEKs were treated with FPP (25, 50, and 100 μg/mL), LG (1, 5, and 25 μg/mL), PH (1, 5, and 25 μg/mL), or NMN (100 μM) for 24 h. NQO1 activity was assessed using an NQO1 Activity Assay Kit and expressed as a percentage relative to that of the untreated control group. Data are shown as mean ± SEM (*n* = 3). Statistical significance was evaluated using one-way ANOVA followed by Tukey’s post hoc test. * *p* < 0.05, ** *p* < 0.01 vs. control.

Measurement of NQO1 activity revealed distinct treatment-dependent effects (Figure 6). In the LG–treated groups, a consistent and statistically significant increase in enzyme activity was observed across all concentrations tested. At 1 μg/mL, LG induced an approximately 30% increase in NQO1 activity compared to that in the untreated control group (*p* < 0.05). At higher concentrations (5 and 25 μg/mL), the magnitude of induction was further elevated, resulting in a 35–40% increase compared to that in the control (*p* < 0.01). These results indicate that LG exerts a robust and reproducible stimulatory effect on NQO1 activity in a concentration-dependent manner.

Similarly, treatment with 100 μM NMN markedly enhanced NQO1 activity. The NMN group displayed an approximately 45% increase compared to that of the control group, which was statistically significant (*p* < 0.01). This strong induction suggests that NMN effectively stimulates antioxidant enzyme activity under the conditions tested.

In contrast, no significant changes in NQO1 activity were detected in either the FPP-treated (25, 50, and 100 μg/mL) or the PH–treated groups (1, 5, and 25 μg/mL). In these groups, NQO1 activity levels remained comparable to those of the untreated control, indicating that neither FPP nor PH significantly influenced this antioxidant enzyme within the tested concentration range.

Collectively, these findings demonstrate that both LG and NMN enhance NQO1 activity, whereas FPP and PH have no measurable effects.

### 3.7. SIRT1, SIRT3 and PGC-1α Expression as Markers of Mitochondrial Function

**Figure 7:**
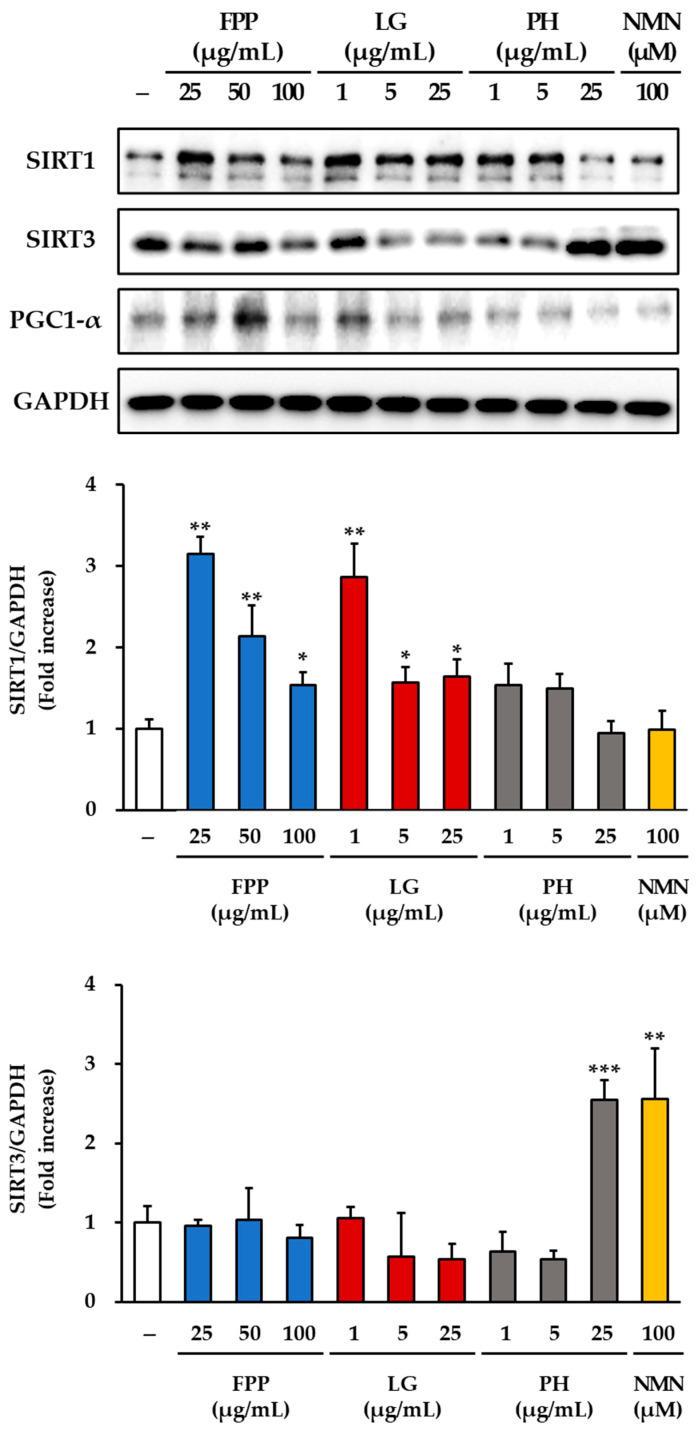

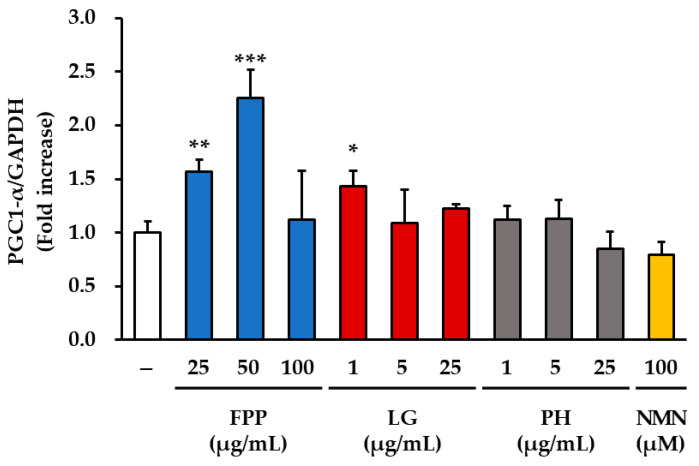
Effects of FPP, LG, PH, and NMN on SIRT1, SIRT3, and PGC-1α protein expression in HEKs. HEKs were treated with FPP (25, 50, and 100 μg/mL), LG (1, 5, and 25 μg/mL), PH (1, 5, and 25 μg/mL), or NMN (100 μM) for 24 h. The protein expression levels of SIRT1, SIRT3, and PGC-1α were determined by Western blotting and normalized to GAPDH. Data are presented as mean ± SEM (*n* = 3). Statistical significance was assessed using one-way ANOVA with Tukey’s post hoc test. * *p* < 0.05, ** *p* < 0.01, *** *p* < 0.001 vs. untreated control.

The expression of sirtuin-related proteins was examined in HEKs treated with FPP, LG, PH, or NMN (Figure 7). For SIRT1, a clear dose-dependent increase was observed in the FPP-treated group compared to that in the untreated control group. Significant upregulation was detected at 25 and 50 μg/mL (*p* < 0.01), with a moderate but statistically significant increase at 100 μg/mL (*p* < 0.05). In the LG–treated groups, SIRT1 expression was elevated, with the strongest effect observed at 1 μg/mL (*p* < 0.01). Significant increases were also observed at 5 μg/mL and 25 μg/mL (*p* < 0.05), indicating that LG consistently promoted SIRT1 expression across all tested concentrations. In contrast, the PH and NMN treatment groups did not show any significant changes in SIRT1 expression compared to the untreated control.

Analysis of SIRT3 expression revealed a different response pattern. No meaningful induction of SIRT3 expression was observed in either the FPP- or LG-treated groups. Only a slight but statistically significant decrease was detected at 25 μg/mL of LG (*p* < 0.05). In contrast, treatment with PH at 25 μg/mL resulted in a marked increase in SIRT3 protein levels (*p* < 0.001), representing the strongest induction among all tested conditions. NMN treatment at 100 μM also significantly enhanced SIRT3 expression (*p* < 0.01), confirming its capacity to activate this mitochondrial sirtuin.

PGC-1α expression was notably upregulated by FPP treatment compared to that in the control group. The greatest increase was observed at 50 μg/mL (*p* < 0.001), followed by a significant increase at 25 μg/mL (*p* < 0.01). LG treatment produced a modest but significant increase in PGC-1α protein expression at 1 μg/mL (*p* < 0.05), whereas no significant changes were detected at higher concentrations (5 and 25 μg/mL). The PH and NMN treatment groups did not show any significant alterations in PGC1α expression compared to that in the control group.

Taken together, these results demonstrate that FPP enhances the expression of SIRT1 and PGC-1α in a concentration-dependent manner, whereas LG stimulates SIRT1 expression across all tested concentrations and modestly upregulates PGC-1α expression at low concentrations. In contrast, PH and NMN had little effect on SIRT1 or PGC-1α expression but strongly induced SIRT3, suggesting the differential regulatory roles of these compounds in mitochondrial and metabolic signaling pathways.

## 4. Discussion

This study explored the anti-senescence potential of fermented porcine placenta (FPP) and its dipeptide constituents, leucine–glycine (LG) and proline–hydroxyproline (PH), in human epidermal keratinocytes (HEKs). Nicotinamide mononucleotide (NMN) was used as a reference compound to target nicotinamide adenine dinucleotide (NAD^+^)-dependent pathways. Multiple cellular parameters, including senescence-associated markers (SA-β-gal, p16, and p21), oxidative stress (ROS), redox balance (NAD^+^/NADH ratio), energy metabolism (ATP production), antioxidant defense (NQO1 activity), and mitochondrial regulatory proteins (SIRT1, SIRT3, and PGC-1α), were assessed to delineate the biochemical responses elicited by each treatment.

FPP markedly attenuated cellular senescence markers at higher concentrations, accompanied by increased ATP synthesis and upregulation of the SIRT1–PGC-1α signaling pathway. These data indicate that FPP may engage multiple intracellular mechanisms associated with mitochondrial function and the maintenance of redox homeostasis. In contrast, LG and PH displayed narrower but distinct activity profiles. LG consistently improved the cellular redox state by suppressing ROS accumulation and elevating the NAD^+^/NADH ratio, whereas PH effectively downregulated p16 and p21 expression and enhanced SIRT3 levels, implying a role in mitochondrial stress response. Although both dipeptides contributed to specific aspects of the observed effects, neither reproduced the entire spectrum of FPP activities. 

Among the tested compounds, LG exhibited the most stable antioxidant action, maintaining a favorable NAD^+^/NADH balance across concentrations. These findings align with previous studies showing that NAD^+^ decline contributes to aging in multiple tissues, including the skin, liver, and brain [16], and that UV exposure accelerates NAD^+^ depletion through DNA repair [17]. In comparison, FPP and PH exhibited significant but concentration-restricted efficacy, suggesting that their activity may depend on the specific intracellular context. As mitochondrial dysfunction and elevated ROS production are pivotal in the aging process, these cellular responses provide mechanistic clues to the potential protective roles of these compounds. Although the precise mechanism remains unclear, PH reduced ROS levels only at 1 μg/mL. This phenomenon may be attributed to PH interacting with a specific intracellular target involved in ROS regulation. Once this target becomes saturated, higher concentrations no longer elicit additional effects, explaining why the reduction is most pronounced at the lowest concentration.

Regarding bioenergetic regulation, FPP promoted ATP generation at all examined concentrations, with a maximal effect at 25 μg/mL, suggesting enhanced mitochondrial activity. LG increased ATP content only at the lowest dose (1 μg/mL), while PH had no measurable influence, implying that its anti-senescence effect may proceed through non-energetic mechanisms. As expected, NMN increased ATP production, consistent with its established function as an NAD^+^ precursor [18,19].

Distinct regulatory signatures were identified in sirtuin-associated pathways. FPP and LG predominantly increased SIRT1 expression, whereas PH and NMN enhanced SIRT3 expression. PGC-1α was strongly induced by FPP and modestly induced by low-dose LG, but remained unchanged following PH or NMN exposure. These findings suggest that FPP primarily modulates the SIRT1–PGC-1α axis, whereas PH and NMN preferentially activate SIRT3-linked mitochondrial programs [22,23,24,25]. LG appears to act at the intersection of these responses by reinforcing both redox balance and SIRT1 signaling, although its effect on ATP production diminished at higher concentrations.

As FPP is a complex mixture containing numerous metabolites, the activities of LG and PH alone cannot fully account for its overall biological effects. Other unidentified constituents may interact synergistically, contributing to the broader cellular responses observed with FPP. In contrast, NMN, being a single small molecule, exhibited more specific but limited NAD^+^-related activity within this experimental framework.

Although the present findings are confined to in vitro keratinocyte assays, they provide preliminary evidence that FPP and its metabolites modulate several cellular pathways related to oxidative stress, energy metabolism, and mitochondrial regulation. Future studies employing three-dimensional skin equivalents and animal models will be essential to validate these mechanisms, establish dose–response relationships, and evaluate their physiological relevance.

Additionally, although NQO1 indirectly regulates ROS levels, its central role in NAD^+^ regeneration and mitochondrial homeostasis justifies its inclusion in this study [30,31]. Nonetheless, future studies should include a broader assessment of antioxidant defenses, such as glutathione-related enzymes, catalase, and peroxidase, to comprehensively elucidate oxidative stress–related mechanisms [32].

In summary, FPP demonstrated the most extensive anti-senescence–associated activity among the tested samples, whereas LG and PH showed distinct mechanistic contributions. While NMN acts primarily through NAD^+^-dependent processes, FPP appears to influence multiple targets involved in mitochondrial and redox regulation. These results highlight FPP as a promising candidate for further investigation into placenta-derived preparations that may support cellular resilience against aging-associated stress in skin cells.

## 5. Conclusions

In conclusion, this study provides experimental evidence that fermented porcine placenta (FPP) exerts notable anti-senescence effects in human epidermal keratinocytes, and its dipeptide derivatives, leucine–glycine (LG) and proline–hydroxyproline (PH), contribute distinct mechanistic roles. FPP treatment led to a reduction in SA-β-gal activity and p16 expression, promoted ATP synthesis, and enhanced the expression of mitochondrial regulators such as SIRT1 and PGC-1α, suggesting its involvement in multiple cellular pathways related to mitochondrial activity and redox regulation.

LG primarily acts as a redox modulator, decreasing intracellular ROS levels, increasing the NAD^+^/NADH ratio, and stimulating antioxidant enzymes, such as NQO1, together with SIRT1 upregulation. In contrast, PH downregulated senescence-associated proteins (p16 and p21), lowered ROS levels at low doses, and markedly increased SIRT3 expression, indicating its possible role in mitochondrial maintenance and stress adaptation.

Nevertheless, the biological activity of FPP cannot be fully explained by LG and PH alone, implying that other components within FPP may interact synergistically to produce a broader cellular response. Compared to NMN, which specifically targets NAD^+^-dependent pathways, FPP exhibited more diverse regulatory effects on oxidative stress, energy metabolism, and mitochondrial signaling under the experimental conditions used in this study.

Overall, these findings suggest that FPP serves as a multifactorial modulator of cellular senescence in keratinocytes, with LG and PH providing partial mechanistic insights into its composite action. Further investigations employing three-dimensional skin equivalents and in vivo systems are warranted to confirm these effects and to evaluate the physiological relevance of placenta-derived bioactive compounds in the context of skin aging.

## Data Availability

The original contributions presented in this study are included in the article. Further inquiries can be directed to the corresponding authors.

## Abbreviations

ATP	Adenosine Triphosphate
AMPK	AMP-activated protein kinase
BCA	Bicinchoninic Acid Assay
DCFDA	2′,7′-Dichlorofluorescin Diacetate
DNA	Deoxyribonucleic acid
FPP	fermented porcine placenta
GAPDH	Glyceraldehyde-3-Phosphate Dehydrogenase
HEKs	Human Epidermal Keratinocytes
IL-1β	Interleukin-1 beta
IL-6	Interleukin-6
LG	Leucine–Glycine
NAD	Nicotinamide Adenine Dinucleotide
NR	Nicotinamide riboside
NADH	Reduced Nicotinamide Adenine Dinucleotide
NMN	Nicotinamide Mononucleotide
NQO1	NAD(P)H Quinone Dehydrogenase 1
P21	Cyclin-Dependent Kinase Inhibitor 1A (CDKN1A)
P53	Tumor Protein P53
P16	Cyclin-dependent kinase inhibitor 2A (CDKN2A)
PBS	Phosphate-Buffered Saline
PGC-1α	Peroxisome Proliferator-Activated Receptor Gamma Coactivator 1-alpha
PH	proline–hydroxyproline
Pla-Ext	Placental Extract
RIPA	Radioimmunoprecipitation Assay Buffer
ROS	Reactive Oxygen Species
SA-β-gal	Senescence-Associated β-Galactosidase
SIRT1	Sirtuin 1
SIRT3	Sirtuin 3
tBHP	tert-Butyl Hydroperoxide
TFAM	Transcription Factor A
UV	Ultraviolet Radiation
